# Progress and promise of CAR-T cell treatment in autoimmune diseases

**DOI:** 10.1371/journal.pmed.1005179

**Published:** 2026-07-20

**Authors:** Jule Bachl, Melanie Hagen, Georg Schett

**Affiliations:** 1 Department of Internal Medicine 3, Rheumatology and Immunology, Friedrich-Alexander-Universität Erlangen Nürnberg and Universitätsklinikum Erlangen, Erlangen, Germany; 2 Deutsches Zentrum Immuntherapie, Friedrich-Alexander-Universität Erlangen Nürnberg and Universitätsklinikum Erlangen, Erlangen, Germany

## Abstract

Chimeric antigen receptor (CAR)-expressing cells bear a great potential for the treatment of autoimmune diseases. In this Perspective, Jule Bachl, Melanie Hagen, and George Schett discuss the challenges and potential of recent technological developments for transforming care for patients with autoimmune disease.

## Teaser

Chimeric antigen receptor (CAR)-expressing cells bear a great potential for the treatment of autoimmune diseases. While numerous challenges persist, recent technological developments have demonstrated the potential for CAR cells to reset dysfunctional immune systems and transform care for patients with autoimmune disease.

## Introduction

**Fig 1:**
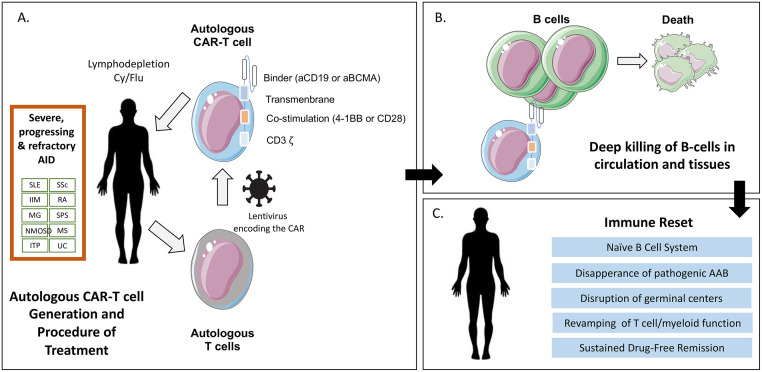
Generation and application of autologous CAR-T cells in autoimmune diseases. **(A)** Generation of CAR-expressing cells by isolation and *ex vivo* expansion of autologous T cells, and transduction by a lentivirus encoding the CAR. These cells are then re-infused in the same patient after lymphodepletion with cyclophosphamide (Cy) and fludarabine (Flu), which allows CAR-T cells to appropriately expand in the patient and establish a population large enough to kill all B cells. The structure of the CAR is shown with specific domains. **(B)** Deep B cell depletion by CAR-T cells. **(C)** Features of immune reset after treatment with CAR-T cells in patients with AID. In the vast majority of patients, B cells reappear at a median time of 3 months, showing a virtually exclusive naïve phenotype, which sustains over many months, or even years [5,6]. GPA, Granuloamatosis with Polyangiitis; IIM, Idiopathic Inflammatory Myopathy; ITP, Idiopathic Thromobocytopenic Purpura; MG, Myasthenia Gravis; MS, Multiple Sclerosis; NMOSD, Neuromyelitis Optica Spectrum Disorder; RA, Rheumatoid Arthritis; SLE, Systemic Lupus Erythematosus; SPS, Stiff Person Syndrome; SSc, Systemic Sclerosis; UC, Ulcerative Colitis.

Chimeric antigen receptor (CAR) T cells are cell-based therapies for the treatment of malignant and autoimmune diseases [1,2]. In contrast to other drugs, such as chemical compounds or monoclonal antibodies, CAR-T cells are “living drugs” conveying the physiological properties of a human immune cell (e.g., migration, activation, proliferation) and combining them with a redirected pharmacological function. This redirected function is based on the expression of the CAR, which allows these therapeutic cells to specifically bind B cells through recognition of the B cell-specific surface molecule CD19 (or alternatively B cell maturation antigen, BCMA) and successively kill target cells (see Fig 1). CAR-T cells are highly effective B cell killers in all human tissues; they not only eliminate circulating B cells but also eradicate the entire pool of tissue B cells in the secondary lymphatic tissues and in target organs affected by autoimmune disease (AID) [3]. This deep depletion—together with a revamping of the entire immune cell landscape after CAR-T cell treatment—has been described as “immune reset”, which clinically manifests in deep and sustained drug-free remission [2]. The most established form of CAR-T cell treatment is autologous therapy [4,5], which is outlined in Fig 1.

Treatment with autologous CAR-T cells targeting CD19 has been approved in B cell malignancies and was first used in AID in 2021, when a patient with refractory systemic lupus erythematosus was treated with CD19 CAR-T cells [6]. Since then, CD19-CAR-T cell treatment has not only rapidly expanded to other autoimmune rheumatic diseases [4,5], but also neuroinflammatory disorders, immune-driven cytopenia and inflammatory bowel disease.

Advantages of CAR-T cell therapy are based on their possibility to eliminate the entire B cell compartment and thereby also autoimmune B cells. By their ability to deeply deplete B cells, CAR-T cells also disrupt germinal center structures, eliminate follicular dendritic cells and block antigen presentation, which affects the T cell and myeloid compartments. This process induces a state of immune homeostasis (“immune reset”; Fig 1C), which needs further characterization but appears to permit sustained remission of AID in the absence of further immune suppression [2]. This effect may have crucial long-term benefits for patients treated with CAR-T cells, i.e., a lower infection risk and higher flexibility for family planning due to the absence of further immune suppression. In addition, the low risk for higher-grade cytokine release syndrome, neurological side effects, and hematotoxicity supports the feasibility of CAR-T cell therapy for AID [7]. However, further data are required to better characterize their long-term safety profile; notably, to date the overall number of published cases on patients with AID treated with CAR-T cells is still limited and further consolidation of their safety and efficacy profile is needed. Key questions concern the emergence of late-onset neutropenia (low numbers of neutrophils) and persistent B cell aplasia (reduction or absence of B cells). Additionally, clinical attention must focus on the long-term risks of infection, secondary malignancies, and cardiovascular disease—conditions that are exacerbated in patients with AID.

## Considerations and questions for CAR-T cell therapy for AID

Despite promising results, the rapid advent of CAR-T cell therapy for AIDs means that several questions remain unanswered. For example, it is currently unknown whether in some patients long-lived plasma cells will need to be eliminated to induce sustained immune reset. These cells are not eliminated by CD19-CAR-T cells, as plasma cells lack expression of CD19. While CAR-T cells targeting BCMA-expressing plasma cells are available and have been successfully used in AID [8–10], eradication of immune memory and the necessity to substitute immunoglobulins in conjunction with BCMA-CAR-T cell therapy poses additional challenges. An additional open question is the level of lymphodepletion that is needed to make CAR-T cell therapy successful. Lymphodepletion regimens, consisting of cyclophosphamide and fludarabine, have been adopted from oncology but have not been developed for AID. Hence, lower-dose lymphodepletion regimens may be feasible and sufficient to allow the expansion of CAR-T cells in vivo.

Considering that CAR-T cell therapy induces a virtually complete B cell depletion in patients, which is followed by reconstitution with a naïve B cell repertoire, responsiveness to vaccination is of critical interest, and may impact the long-term safety of this approach. While no humoral immune response to vaccination can be expected in the B cell aplasia phase, preliminary data suggest normal humoral immune responses after B cell reconstitution [4]. However, structured assessment of vaccination with de novo and recall antigens will be necessary to define vaccination responses in a situation where the memory B cell pool is substantially contracted. Furthermore, while CD19-targeted CAR-T cells do not target long-lived plasma cells and leave most existing vaccination responses intact [5,6], BCMA-targeted CAR-T cells also deplete long-lived plasma cells and therefore interfere with existing humoral vaccination responses. Hence, re-vaccination may be obligatory after BCMA-targeted CAR-T cell therapy, and its efficacy will need to be investigated.

Potential barriers for autologous CAR-T cell treatment include the high manufacturing costs and the limited number of advanced treatment centers. Nonetheless, some of these barriers have been overcome in oncology by more accurate selection of high-risk patients, hospital exemption programs and advanced CAR-T cell production methods. Similar developments are currently seen in the treatment of patients with AID with autologous CAR-T cells. Furthermore, medical costs have shown to decrease by more than 90% after successful CAR-T cell therapy in AID, which results from a sharp decrease in drug therapy, lower need for inpatient and outpatient management of complications, and a diminished requirement of rehabilitative and organ-support measures [11]. Considering that the majority of patients with AID treated with CAR-T cells indeed maintain drug-free remission over years, the benefits of this treatment may outweigh the costs.

## Can new technologies pave the way to the clinic?

New technologies are rapidly evolving. These include allogeneic CAR-T cells, which are usually produced from inducible pluripotent stem cells (iPSCs) differentiated into T cells or natural killer (NK) cells expressing a CAR. These “off-the-shelf” cell therapy products are gene-edited to remove critical antigenic surface structures in order to prevent their immediate rejection, as well as to reduce graft-versus-host disease. Allogeneic CAR-T cell products can be readily used in larger patient populations and do not require individual cell manufacturing. Meanwhile, a very recent approach is in vivo CAR-T cell therapy, whereby CAR-T cells are formed directly in the patient by infusing targeted lipid nanoparticles that contain the RNA payload for the CAR. Once administered, these nanoparticles fuse with target cells (usually T cells) and induce the expression of the CAR. Killing of B cells then functions in a similar way as with *ex vivo* CAR-T cell therapy. While the principal feasibility of allogeneic and in vivo CAR-T cell therapy in AID has been demonstrated [12,13], it is currently unclear whether these approaches can induce sustained immune reset in patients and allow long-term drug free remission.

A transient mRNA-based CAR expression is principally well suited for its use in AID, avoiding genomic integration and ascertaining reversibility of the CAR expression. In fact, *ex vivo* mRNA-based CAR-T cells have shown clinical efficacy in myasthenia gravis [8]. In the context of in vivo approaches, dimension and durability of CAR expression need to be sufficiently high enough to achieve complete B cell depletion and thereby trigger an immune reset. As CAR expression is diluted with each cell division in mRNA-based expression approaches—showing no genomic integration—upfront generation of sufficient numbers of CAR-T cells seems to be critical, while the proliferation of existing CAR-T cells in vivo, which is the most powerful process in autologous *ex vivo* CAR-T cell therapy, seems less important. Repeated injection of targeted mRNA-CAR carrying lipid nanoparticles, as well as vector-based in vivo CAR-T cell therapies, may overcome these issues. Vector-based in vivo therapies are in development, but require stringent safety considerations due to in vivo DNA integration. Overall, these new methods will push forward the CAR-T cell field and eventually allow improved scalability of treatment.

## Conclusions

With the first autoimmune patient reaching 5 years drug-free remission after a single infusion of CAR-T cell in March 2021, cure of AID has become within reach for the first time. It is widely accepted that cancer can be cured and a 5-year cancer-free survival is used as a benchmark by the National Cancer Institute’s Surveillance, Epidemiology, and End Results Program [14]. Further data are necessary to support the concept of cure after more than 5 years absence of AID; however, with the growing insights from CAR-T cell therapy, a gradual shift of therapeutic expectations from remission to cure will likely occur. Based on the preliminary but encouraging safety and efficacy data, several pivotal studies with autologous CAR-T cells—alongside early studies with allogeneic CAR-T and CAR-NK cells, as well as in vivo CAR-T cells—are currently underway, which will considerably extend the knowledge on this new therapy. It is yet too early to say where these treatments are best placed in the treatment landscape, but it is likely that patients with severe forms of AID or those showing inadequate responses to conventional treatments are the ones who may profit the most. Identification of such patient populations will be critical in order to initiate timely treatment before substantial irreversible damage accrues.

## Abbreviations

AID: autoimmune disease
BCMA: B cell maturation antigen
CAR: chimeric antigen receptor
iPSCs: inducible pluripotent stem cells
NK: natural killer
